# Great Malformation of the Heart; a Single Auricle and Ventricle

**Published:** 1845-12

**Authors:** 


*Great Malformation of the Heart; a single Auricle and Ventricle.*—During the first six weeks of life, the child (the subject of the present case) seemed to thrive perfectly well; but then the breathing became difficult, and the surface of the face and body to exhibit a bluish hue. At six months, she was seized with convulsions, which were followed by hemiplegia of the right side. This, however, gradually became less and less, and eventually the young sufferer recovered so well, that she could walk about with ease, after the right *tendo Achilles* (which had become contracted) was divided by M. Scoutteten. The dyspnœa and cyanosis were always increased upon any exertion; the blue tint was more *prononcé* on the right side. In her sixth year the girl died of an attack of bronchitis. *Dissection.*—The substance of the two ventricles of the heart was nearly of the same thickness throughout. The septum was almost entirely wanting, there being no trace of it except at the lower part. The orifice of the pulmonary artery was separated from that of the aorta only by a small spur, which formed the upper part of the circumference of the inter-ventricular opening. There was only one auriculo-ventricular orifice, common to the two ventricles and two auricles. These last-named cavities were separated from each other by a thin septum, which did not reach as far as this orifice, and therefore was incomplete. The *foramen Botalii* also was so open as to admit the point of the little finger. Thus it was that a free communication existed not only between the cavity of the ventricles and that of the auricles, but also from one auricle to the other. The auriculo-ventricular orifice was provided with a large triangular valve, that was attached to the anterior three-fourths of its circumference, and fixed at its apex to the *columnæ carneæ* on the posterior part of the ventricular parietes. A few *columnæ*, proceeding from both ventricles, were attached to the two lateral borders of the valve.

In this case therefore, although there were distinct vestiges of four cardiac cavities, we may fairly say that the heart was simple—*i. e.* consisting of one ventricle and one auricle—as it exists in Bratachian animals. The presence of a single auriculo-ventricular orifice can leave no doubt on this point.—*Med. Chir. Rev. from Gaz. Med.*

